# Bisphenol A Levels in Pasteurized Milk Marketed in Plastic Packaging and Associated Health Risk Assessment: A Pilot Study

**DOI:** 10.3390/jox15060180

**Published:** 2025-11-01

**Authors:** El Amine Cheroual, Khatima Mezhoud, Ilaria Neri, Ouahiba Hadjoudj, Lucia Grumetto

**Affiliations:** 1 Laboratory of Hydrology and Bromatology, Department of Pharmacy, Faculty of Medicine, University Ferhat Abbas Setif 1, Setif 19137, Algeria; elamine.cheroual@univ-setif.dz; 2 Laboratory of Medical Botany and Cryptogamy, Department of Pharmacy, Faculty of Medicine, University Salah Boubnider 3, Constantine 25016, Algeria; pharmabac12@hotmail.fr; 3 Department of Pharmacy, School of Medicine and Surgery, University of Naples Federico II, Via D. Montesano, 49, I-80131 Naples, Italy; ilaria.neri@unina.it; 4 Laboratory of Hydrology and Bromatology, Faculty of Pharmacy, University of Health Sciences, Algiers 16000, Algeria; ohadjoudj@yahoo.fr

**Keywords:** plastic packaging, bisphenol A, pasteurized milk, risk assessment, daily intake

## Abstract

Bisphenol A (BPA) is a synthetic estrogen widely used in the manufacture of food packaging materials, raising concerns due to its potential migration into food products. This study aims to determine BPA levels in pasteurized milk marketed in Algeria, using an easy-to-handle and efficient liquid–liquid extraction method coupled with liquid chromatography and fluorescence detection. A total of 30 pasteurized milk samples packaged in plastic were analyzed. The method validation demonstrated excellent linearity, with a limit of detection of 3.76 µg/L and a limit of quantification of 11.40 µg/L. Among the analyzed samples, 17 contained detectable BPA levels, ranging from not detectable to 24.07 µg/L, with an average concentration of 3.77 ± 5.77 µg/L, compliant with European regulation. The health risk assessment, based on estimated chronic daily intake and hazard index, indicated no significant risk associated with BPA exposure through milk consumption in the studied population. Additionally, the estrogenic equivalence of BPA in milk was 6.032 × 10^−5^ µgE_2_/L, confirming a low estrogenic activity.

## 1. Introduction

Bisphenol A (BPA) is a monomer widely used in industrial manufacturing, particularly in the production of polycarbonate plastics and epoxy resins intended for direct contact with food [1,2]. BPA, found to act as an endocrine disruptor, can migrate from packaging into the food both during high-temperature processes such as sterilization and if the packaging is damaged [3,4]. BPA migration levels can also be significantly influenced by the shelf life of the product and, therefore, the contact time, but also by manufacturing methods, type of canned food (salty, sweet, or high-fat), the presence of mineral elements in the food, and repeated use or aging of cans [4,5]. In addition, BPA migration is more significant if the food is in contact with epoxy resins than in food with plastic packaging [6]. The widespread occurrence of BPA in canned foods raises significant concerns because the diet is the main route of human exposure [7,8]. BPA exhibits estrogenic properties, although less powerful than estradiol-17β, and it has been related to various health risks, such as reproductive disorders, obesity, diabetes, and alterations to neurodevelopment in fetuses [9,10,11,12]. The European Union (EU) has implemented stringent regulations on the use of BPA in food-contact materials, setting firstly a specific migration limit of 0.05 µg/L in food products [13]. Similarly, the U.S. Environmental Protection Agency (USEPA) has established a tolerable daily intake (TDI) of 50 µg/kg of body weight per day [14]. However, following experts’ report from the European Food Safety Authority (EFSA), which highlighted BPA’s adverse effects on the immune system, a significantly lower TDI of 0.2 ng/kg of body weight per day was introduced by the EU [15]. A more recent regulation adopted a ban on BPA use in food-contact materials, on December 2024, the European Commission published Regulation (EU) 2024/3190, on the use of BPA and bisphenols analogs and derivatives with a harmonized classification for specific hazardous properties in certain materials and articles intended to come into contact with food; such regulation amends regulation (EU) No 10/2011 and repeals regulation (EU) 2018/213, prohibiting the use of BPA and its salts in all materials intended to come into contact with foodstuffs, including plastics, varnishes, coatings, and related substances. The regulation came into force in January 2025, representing an important milestone in the EU’s efforts to reduce consumer exposure to hazardous chemicals. The decision approved by Member States will take effect in July 2026. The ban specifically targets the inner linings of metal cans and refillable plastic bottles [16]. Despite regulatory restrictions imposed by the EU and other countries, based on documented health risks, particularly for infants and young children, whose developmental impacts may be irreversible and persist throughout their lives, BPA remains widely present in many everyday consumer products [17,18]. BPA has been found in various types of foodstuffs throughout the world, at concentrations that sometimes exceed the European standard of 50 μg/kg [19]. Milk and dairy products represent a possible source of exposure to BPA, particularly for young children, both due to its migration from food packaging and possible contamination of the milk food chain [20,21]. In fact, BPA can accumulate in the adipose tissue of dairy cows and then be excreted in their milk [21,22]. Concentrations of BPA in dairy products can vary as a function of several factors, including the type of product (fresh or canned), sterilization processes, and the season, underlining the need for continuous monitoring of its levels in milk [23]. Although there is an increasing concern about its presence in foodstuffs, data on BPA contamination of milk in Algeria, and more widely in Africa, remain limited or even lacking [24]. Pasteurized milk is one of the most widely consumed products in Algeria and an essential source of nutrition for the population. Therefore, the aim of this study was to determine BPA levels in pasteurized milk marketed in Algeria using a simple and efficient extraction method, as well as rapid detection by liquid chromatography coupled with fluorescence detection (HPLC/FLD). This analytical method can (i) enable easy and inexpensive determination of BPA in dairy matrices, to assess the health risks associated with the possible presence of BPA in milk, and (ii) improve its monitoring and prompt Algerian authorities to take action and set regulatory thresholds similar to those adopted by EU and non-EU countries.

## 2. Materials and Methods

### 2.1. Reagents and Chemicals

BPA (≥99%), potassium ferrocyanide (≥99%), zinc acetate (≥99%), lead acetate (≥99%), and acetonitrile (ACN) (high-performance liquid chromatography (HPLC)-grade solvents) were acquired from Sigma-Aldrich (Milan, Italy). Ultra-purified water (Milli-Q) was produced in-house, with a conductivity of 0.055 μS/cm at 25 °C and resistivity of 18.2 MΩ·cm. BPA stock solutions (2 mg/mL) were prepared in ACN, and standard solutions were prepared by mixing and diluting the stock solutions with ACN. Potassium ferrocyanide (15%), zinc acetate (30%), and lead acetate (30%) solutions were diluted with ACN. All prepared solutions were kept at 4 °C and used within 3 months. The mobile phases were filtered under vacuum using 0.22 μm nylon membranes (Millipore, Burlington, MA, USA and Sigma-Aldrich, Milan, Italy).

### 2.2. Sample Collection and Preparation

Milk samples (30) from four different brands, all packaged in plastic, were purchased from local markets in northern Algeria, where most of the population lives and milk consumption is highest. The samples consisted of eight from one brand collected in the west, eight from another brand in the center, and fourteen from the east (two brands with seven samples each). This selection was intended to reflect the main areas of milk production and consumption in Algeria. This African country represents a critical case study for assessing potential exposure risks, as dairy consumption is widespread and specific national regulations governing BPA contamination in food products are limited.

The sample preparation procedure was adapted from Ballesteros-Gómez et al., with some modifications [25]. Each sample comprised three one-liter bags from the same production batch. The bags were made of high-density polyethylene. The samples were prepared by mixing the three bags; then, 20 mL of milk was mixed with (i) 2 mL of potassium ferrocyanide, (ii) 2 mL of zinc acetate, and (iii) 2 drops of lead acetate. The volume was made up to 100 mL with deionized water. The mixture was then homogenized, sonicated at 40 kHz at room temperature using an ultrasound apparatus (Sonorex^®^, Bandelin Electronic GmbH & Co. KG, Berlin, Germany), followed by filtration through 0.45 µm polyamide syringe filters (DORSAN®, Dorsan Filtration S.L., Vilafranca del Penedès, Spain). The filtered solution was diluted to 200 mL with deionized water. 50 mL of the dilution was concentrated to approximately 2 mL using a rotary evaporator (BUCHI®, R-100, Büchi Labortechnik AG, Flawil, Switzerland) and then evaporated to dryness using a Dry Vacuum Concentrator (Christ®, RVC 2-18 CDplus, Martin Christ Gefriertrocknungsanlagen GmbH, Osterode am Harz, Germany). The dry residue was dissolved with 1 mL of ACN before analysis by liquid chromatography.

### 2.3. Chromatographic Analyses

The chromatographic system (LC-20AD, Shimadzu Corp., Kyoto, Japan) was fitted with a reversed-phase Ascentis^®^ C18 stainless-steel column (250 mm × 4.60 mm i.d., 5 µm) and a Supelguard Ascentis^®^ C18 guard column (Supelco, Bellefonte, PA, USA). Detection was carried out using an SPD-20A fluorescence detector (Shimadzu Corp., Kyoto, Japan) operating at an excitation wavelength of 273 nm and an emission wavelength of 303 nm. The mobile phase contained acetonitrile and water (60:40, *v*/*v*). The analysis was performed at room temperature (20 °C) with a flow rate of 0.5 mL/min in isocratic mode, and the injection volume was 60 µL. Fluorescence data were acquired and processed using Chromatoplus software® 2011 (Shimadzu Corp., Kyoto, Japan). BPA retention time was 9.80 min. Each sample was analyzed in duplicate [2].

### 2.4. Method Validation

Three glass-packed milk samples were analyzed for the absence of detectable BPA and chromatographic interferences. They were then spiked, respectively, with 200 µL, 50 µL, and 10 µL of a standard BPA solution (2 mg/mL) to assess extraction recovery at high (400 µg/mL), medium (100 µg/mL), and low (20 µg/mL) concentrations. Samples were then subjected to the extraction procedure described above. Extraction efficiency at each concentration was determined by comparing the mean peak area of the spiked sample before extraction to that of the corresponding reference standard solution. The validation process included the assessment of linearity, limit of detection (LOD), limit of quantification (LOQ), and precision (repeatability) within the milk matrix. For quantitative determinations, matrix-matched calibration curves were employed, and the HPLC system was also calibrated using standard BPA solutions in acetonitrile at concentrations of 12.5, 25, 50, 100, 200, and 400 µg/L. The LOD and LOQ values were estimated using the formula 3.3 σ/S and 10 σ/S, respectively, where σ represents the standard deviation of the response and S denotes the slope of the calibration line. Intraday repeatability was assessed by carrying out ten injections of standard BPA solutions at a concentration of 12.5 µg/L. The validation parameters were evaluated according to the recommendations of the ICH Q2(R2) guidelines [26].

### 2.5. Health Risk Assessment Based on Chronic Daily Intake and Hazard Index

The assessment of health risks associated with BPA exposure was conducted in accordance with the USEPA and EFSA guidelines [14,15]. In this study, the potential risks associated with BPA ingestion through pasteurized milk packaged in plastics were assessed for men and women in four age categories: 6–13 years, 14–19 years, 20–27 years, and 27–60 years. Data were collected in various Algerian cities to ensure representativeness [27].

#### 2.5.1. Chronic Daily Intake

The exposure to BPA via milk consumption was quantified by calculating the Chronic Daily Intake (CDI) according to the following equation:

CDI = C × IR/BW(1)

C is the average BPA concentration in milk (μg/L);

IR represents the daily ingestion rate of milk (L/day);

BW refers to the mean body weight of individuals within each age group (kg).

The CDI provides an estimate of the daily intake of BPA per kilogram of body weight, which is a crucial parameter for health risk assessment [28]. Reference values for intake rates and body weights specific to each age group are reported in Table 1.

#### 2.5.2. Risk Characterization

To characterize the non-carcinogenic risk associated with BPA oral exposure, the hazard index (HI) was computed using the following formula:

HI = CDI/RFD(2)

CDI represents the chronic daily intake (μg/kg/day);

RFD is the reference dose for BPA, established at 50 μg/kg/day according to the USEPA, and at 0.2 ng/kg/day according to the EFSA [14,15].

An HI value below 1 indicates that exposure is unlikely to cause adverse health effects, while an HI value above 1 suggests a potential risk that warrants further investigation.

#### 2.5.3. Assessment of Estrogenic Activity

Although BPA displays significantly lower estrogenic activity compared to endogenous estrogens, elevated concentrations in food matrices may potentiate its endocrine-disrupting effects. The estrogenic activity of BPA was quantified through the calculation of Estrogen Equivalency (EEQ), using the following formula:

EEQ = EP × C(3)

C represents the average concentration of BPA (μg/L) in milk;

EP represents the estrogenic potency of BPA, set at 0.0000160.

EEQ values are expressed in μgE_2_/L, with 17β-estradiol (E_2_) selected as the reference compound (EP = 1), due to its potent estrogenic properties. Thus, compounds with an EP lower than 1 demonstrate weaker estrogenic activity compared to E_2_ [28,29].

## 3. Results

### 3.1. Method Validation Results

Each concentration level of the calibration curve was repeated five times, and the calibration was repeated every two weeks. The coefficient of variation remained below 0.5%. Solutions were analyzed by HPLC/FLD immediately after preparation, with no detectable degradation products. The method revealed excellent linearity, with a correlation coefficient (r^2^) of 0.9999. The LOD and LOQ were 3.76 µg/L and 11.40 µg/L, respectively. Intraday precision, evaluated by relative standard deviation (RSD%), was 12.63%, in accordance with EU 11312/2021 guidelines, which recommend a percentage below 20% [30]. Recovery levels ranged from 84.92% to 104.28%. Validation parameters are summarized in Table 2.

To evaluate BPA levels in milk packaged in plastic containers, we applied the validated method to the analysis of real milk samples. The identification of the BPA peak was performed by comparison with a standard solution to confirm the retention time, while quantification was performed based on the signal intensity (Figure S1). To estimate the average BPA concentration in the milk samples, values below the LOD were considered zero, while those below the LOQ were substituted with half the LOQ value [31].

The results of the BPA quantification are shown in Table 3. BPA was detected in 17 out of the 30 analyzed samples (56.67%), with concentrations ranging from not detected (ND) to 24.07 μg/L, and an average concentration of 3.77 ± 5.77 μg/L. All detected values were below the European-specific migration limit of 50 µg/L applicable for food contact materials, applicable until July 2026.

A noticeable variability between brands was observed. Brand 2 showed the highest BPA concentration (24.07 µg/L), while Brand 4 exhibited the highest detection frequency, with five out of seven samples containing detectable BPA levels. Brand 1 showed the lowest contamination, with BPA detected in only three samples. The differences between brands may be attributed to variations in the quality of packaging materials and manufacturing conditions. The variations observed between different batches of the same brand could be related to the quality of the powdered milk used as a raw material.

### 3.2. Health Risk Assessment

The estimated CDI values for BPA were calculated for four age groups: 6–13 years, 14–20 years, 20–27 years, and 27–60 years, with respective values of 0.0261, 0.0123, 0.0069, and 0.0073 μg/kg/day. For both sexes, CDI was estimated as 0.0114 μg/kg/day for men and 0.0084 μg/kg/day for women. In all cases, the BPA CDI values were far below the standard threshold set by the USEPA at 50 μg/kg body weight/day [14]. However, when compared with the newly set TDI by the EFSA at 0.2 ng/kg body weight/day, the measured values greatly exceed this limit, suggesting a potential exposure risk [15]. The non-carcinogenic risk was assessed using the HI, and the results are presented in Table 4. When the HI was calculated using the USEPA reference dose, all values were below 1, ranging from 0.00014 to 0.00052 across age groups and from 0.00017 to 0.00023 for females and males, respectively, indicating exposure levels considered safe under USA standards. In contrast, when calculated using the EFSA reference dose, HI values ranged from 34.5 to 130.5 across age groups and from 42 to 57 for females and males, respectively, all exceeding 1. These results indicate that BPA exposure from milk samples meets the USEPA safety limits but exceeds the newly established EFSA threshold. Finally, the calculated EEQ was 0.000060 μgE_2_/L, indicating that the BPA concentration in milk induces a very low estrogenic activity compared with natural estrogens.

## 4. Discussion

In this study, BPA was detected in 17 out of 30 milk samples packaged in plastic, with concentrations ranging from ND to 24.07 μg/L and an average value of 3.77 ± 5.77 μg/L, well below the European limit of 50 μg/L. These findings are consistent with several reports from the literature, which collectively highlight the widespread occurrence of BPA contamination in commercial milk worldwide. For instance, Kamal et al., in Pakistan, detected BPA in 12 out of 23 milk samples, with concentrations ranging from ND to 56 µg/L. The highest BPA levels (0.056 and 0.042 µg/mL) were found in raw milk stored in polycarbonate containers under high temperatures, showing a clear relationship between temperature and BPA migration. This confirms that BPA leaching varies depending on the type of packaging used for milk [28]. Similarly, in Italy, Grumetto et al. detected BPA in 20 of 68 samples, with concentrations ranging from ND to 521.0 ng/mL [2]. In Brazil, Soares et al. found BPA in 18 commercial samples, ranging from 77.6 to 150.8 ng/mL [32]. In Egypt, Osman et al. reported average BPA levels of 89.35 ng/mL in infant formula and 33.43 ng/mL in condensed milk [33]. In China, Cheng et al. found an average BPA level of 127.2 ng/mL, while in Iran, levels ranged from 9.6 to 23.5 ng/mL [29,34]. These findings collectively affirm that the presence of BPA in milk is a global phenomenon that can be caused by multiple factors, including polymer type, thermal treatment, storage conditions, and duration of contact between milk and packaging material. In addition, contamination can occur at different stages, either through the environment, particularly when animals live in areas where water and soil are polluted by industrial activities, or during processing in factories, through contact with production and storage equipment [35]. The relatively low BPA concentrations detected in this study may be explained by the short shelf life of pasteurized milk sold in plastic packaging, which typically does not exceed three days. This limited contact time reduces the opportunity for BPA migration. Additionally, the moderate heat treatment applied during pasteurization (usually below 80 °C) is unlikely to cause significant polymer degradation or enhance BPA release into milk.

The CDI values for the different age groups included in our study, as well as for both sexes, were all significantly lower than the USEPA threshold of 50 μg/kg/day. Similarly, the calculated HI values remained well below the safety limit of 1, suggesting a negligible non-carcinogenic risk. Likewise, the EEQ was very low (0.000060 μgE_2_/L), indicating minimal endocrine activity. These results are consistent with the global trend, where CDI values generally remain below 1 μg/kg/day of body weight per day [28]. In Pakistan, CDI of BPA in milk samples ranged from 1.42 to 2.67 μg/kg/day and from 5.58 to 10 μg/kg/day, remaining well below the regulatory limit [36]. Conversely, in Iran, the reported CDI values were significantly higher among infants: 191.1, 161.37, and 153.76 μg/kg/day for the age groups 0–6 months, 6–12 months, and 12–24 months, respectively. These values far exceeded the USEPA threshold of 50 μg/kg/day, thus highlighting critical exposure risks during the early stages of life [29]. In a related study conducted in Pakistan, Kamal et al. assessed the HI for milk stored in baby bottles from six brands. Their results indicated that five brands maintained BPA exposure below the safety limit, but one brand exceeded this threshold, potentially posing a health risk to infants aged 0–6 months and 7–12 months. One brand exhibited the highest potential for estrogenic effects, with a value of 0.000032 μgE_2_/L. Three brands showed estrogenic activity levels ranging from 0.0000160 to 0.00000064 μgE_2_/L. In contrast, no estrogenic activity was detected in two brands, as their EP was zero [31].

In this study, we chose to discuss our results with reference to the USEPA guideline rather than the recent EFSA TDI. This decision was motivated by the fact that Algeria has not yet established specific regulatory standards for BPA exposure. Therefore, referring to the USEPA value provides a more suitable and comparative framework for interpreting our findings in the current national context.

Overall, based on the broader standard established by the USEPA, the BPA levels in milk samples analyzed in the present study can represent minimal health risks for the general population. However, it remains crucial to consider vulnerable groups, particularly young children, when evaluating the potential endocrine-disrupting effects associated with BPA exposure even at low doses.

Despite its relevance, this study has some limitations. First, the sample size was relatively limited (30 milk samples), although the dataset still supports our conclusions. Second, seasonal variations and environmental factors that may influence BPA migration were not evaluated. Third, we focused on BPA, while other endocrine disruptors (EDCs), among which bisphenol analogues are used as BPA substitutes, were not included. Considering that these compounds may possess comparable or even greater endocrine-disrupting potential, their exclusion may underestimate the total exposure risk. Additionally, this study did not account for cumulative exposure through other dietary sources, such as canned foods, beverages, or thermal paper receipts, which could contribute to overall BPA body burden.

Future research should adopt a more comprehensive approach by expanding sampling to different milk types (raw, pasteurized, UHT, and infant formula), various packaging materials, and multiple storage conditions to better understand BPA migration dynamics. Moreover, research into the combined effects of other EDCs is crucial to assess the cumulative and synergistic effects on human health.

In developed countries, stringent regulations, advanced packaging technologies, and improved food safety monitoring systems have contributed to lower BPA exposure levels. In developing countries, however, the risk is exacerbated by limited regulatory enforcement, inadequate awareness among consumers and producers, and improper storage practices (such as high-temperature exposure during transport or retail display). The rapid urbanization and industrialization observed in many developing regions further increase environmental BPA contamination, which can bioaccumulate in food chains and pose chronic exposure risks.

To mitigate BPA exposure and safeguard public health, several strategies can be implemented. From a regulatory perspective, governments should establish and enforce strict standards for allowable BPA levels in food contact materials and encourage the transition toward safer, BPA-free packaging alternatives such as glass. From a societal standpoint, educating consumers about safe food storage practices can significantly reduce exposure.

Additionally, investing in public health surveillance and exposure assessment studies, particularly for vulnerable populations, is vital to implement preventive measures.

## 5. Conclusions

This study provides, for the first time, a comprehensive assessment of BPA contamination in milk sold in Algeria, highlighting its occurrence in more than half of the analyzed samples. Although the detected concentrations remained below international safety thresholds, the presence of BPA in milk raises concerns regarding chronic low-dose exposure, and its possible synergistic interactions with other endocrine disruptors, as “a cocktail effect”, combined and potentially amplified, due to the occurrence in the human body of multiple endocrine disruptors not considered harmless individually. The analytical approach employed proved to be highly sensitive and reliable, offering a robust tool for regulatory monitoring. The health risk assessment, including CDI, HI, and EEQ evaluations, indicated no acute toxic health hazard. However, given the ongoing scientific debate on the endocrine-disrupting potential of BPA even at low doses, continuous monitoring remains imperative. These data reflect the analyzed milk samples, and due to the variability in BPA concentrations influenced by environmental contamination and manufacturing processes, systematic analyses of milk samples are necessary. Future research should broaden the scope to include different dairy products, alternative packaging materials, and potential sources of BPA contamination along the production chain. Additionally, considering the new TDI of 0.2 ng/kg/day established by EFSA, as well as the recent ban on BPA in food contact material imposed by EU authorities, the investigation of consumer exposure from multiple dietary sources, also in non-EU and North American countries, can provide a more accurate risk assessment, driving future regulatory policies.

## Figures and Tables

**Table 1:** Reference values for the calculation of chronic daily intake and hazard index.

	Groups	IR (L)	Body Weight (kg)
Sex	Male	0.1875	62
	Female	0.1225	55
Age (years)	06–13	0.235	34
	14–19	0.18	55
	20–27	0.115	63
	27–60	0.142	73

**Table 2:** Validation parameters for the determination of BPA in milk.

Validation Parameters	Values
Range (µg/L)	12.5–400
Slope	47.99
Intercept	−199.473
Linearity (r^2^)	0.9999
RSD %	12.63
LOD (μg/L)	3.76
LOQ (μg/L)	11.40
Recovery (%)	84.92–104.28

**Table 3:** BPA concentrations in milk.

Brand	Sample No.	Concentration (µg/L)
Brand 1	1	7.62 ± 0.15
	2	ND
	3	11.55 ± 0.01
	4	ND
	5	7.96 ± 0.11
	6	ND
	7	ND
	8	ND
Brand 2	9	ND
	10	5.41
	11	ND
	12	2.45 ± 0.77
	13	<LOQ
	14	ND
	15	24.07 ± 0.11
	16	<LOQ
Brand 3	17	ND
	18	ND
	19	<LOQ
	20	4.59 ± 0.06
	21	ND
	22	15.95 ± 0.19
	23	2.34 ± 0.10
Brand 4	24	ND
	25	ND
	26	5.25 ± 0.42
	27	3.51 ± 0.08
	28	13.58 ± 0.33
	29	2.72 ± 0.09
	30	2.57 ± 0.03

**Table 4:** Chronic daily intake and hazard index of milk for different groups.

	Groups	CDI (μg/kg/day)	HI (EFSA)	HI (USEPA)
Sex	Male	0.0114	57	0.00023
	Female	0.0084	42	0.00017
Age (years)	06–13	0.0261	130.5	0.00052
	14–19	0.0123	61.5	0.00025
	20–27	0.0069	34.5	0.00014
	27–60	0.0073	36.5	0.00015

## Data Availability

The original contributions presented in this study are included in the article. Further inquiries can be directed to the corresponding authors.

## Supplementary Materials

The following supporting information can be downloaded at: https://www.mdpi.com/article/10.3390/jox15060180/s1, Figure S1: (a) HPLC/FD chromatogram of the BPA standard (200 µg/L); (b) HPLC/FD chromatogram of a milk sample showing a positive detection of BPA.

## Abbreviations

The following abbreviations are used in this manuscript:

BPA	Bisphenol A
EU	European Union
USEPA	U.S. Environmental Protection Agency
TDI	Tolerable Daily Intake
EFSA	European Food Safety Authority
HPLC/FLD	High Performance Liquid Chromatography coupled with Fluorescence Detection
ACN	Acetonitrile
LOD	Limit Of Detection
LOQ	Limit Of Quantification
CDI	Chronic Daily Intake
C	Concentration
IR	Ingestion Rate
BW	Body Weight
HI	Hazard Index
RFD	Reference Dose
EEQ	Estrogen Equivalency
EP	Estrogenic Potency
r^2^	Correlation Coefficient
RSD	Relative Standard Deviation
ND	Not Detected
EDCs	Endocrine Disruptors
UHT	Ultra-High Temperature
