# Supplementary material for: Bisphenol A Levels in Pasteurized Milk Marketed in Plastic Packaging and Associated Health Risk Assessment: A Pilot Study
Source: J Xenobiot. 2025 Nov 1;15(6):180. doi: 10.3390/jox15060180 (PMC12641992; doi:10.3390/jox15060180)
Supplement: Supplementary file 1 [file jox-15-00180-s001.zip › jox-3918493-supplementary.pdf]

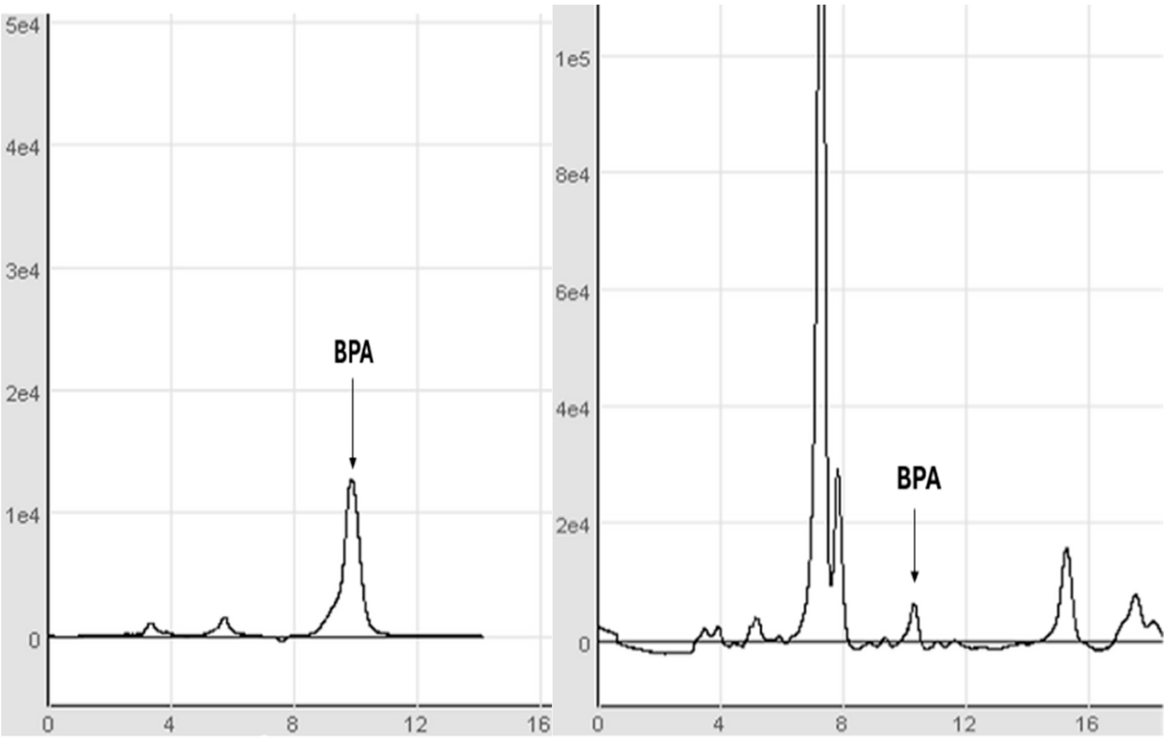


(a) (b)

**Figure S1.** (a) HPLC/FD chromatogram of the BPA standard (200 μg/L); (b) HPLC/FD chromatogram of a milk sample showing a positive detection of BPA.
